# Analyzing the Effect of Drainage on the Stability of Tailings Dams Using the Interpretation of Cross-Correlations

**DOI:** 10.3390/s25061833

**Published:** 2025-03-15

**Authors:** Moustafa Hamze-Guilart, Lineu Azuaga Ayres da Silva, Anna Luiza Marques Ayres da Silva, Maria Eugenia Gimenez Boscov

**Affiliations:** 1 Structures and Geotechnical Engineering Department, Universidade de São Paulo Escola Politécnica, São Paulo 05508-070, Brazil; moustafa@alumni.usp.br (M.H.-G.); meboscov@usp.br (M.E.G.B.); 2 Mining and Petroleum Engineering Department, Polytechnic School, University of São Paulo, São Paulo 05508-030, Brazil; linayres@gmail.com

**Keywords:** tailings dams, geotechnical monitoring, time series, autocorrelation function, cross-correlation, effect of drainage

## Abstract

Over the years, multiple tailings dam failures all over the world have been primarily linked to drainage issues. Given its critical role in dam stability, this research analyzes the relationship between precipitation, reservoir levels, and geotechnical instrumentation measurements along the elevation stages of a tailings dam. To assess the influence of drainage on dam performance, its dependence on infiltration, reservoir water fluctuations, and geotechnical instrumentation responses was modeled and interpreted. By applying time series analysis methods to the instrumentation data, including autocorrelation and cross-correlation functions, this study identifies patterns in drainage efficiency and its impact on stability. The time series data were regularized and transformed into stationary forms to ensure consistency in the analysis. Autocorrelation functions and cross-correlations between different monitoring instruments were computed specifically for the second to the seventh elevation stages of the tailings dam. This study focuses on four cross-sections of the dam, analyzing their behavior to differentiate the spatial and temporal effects of drainage. The results reveal variations in drainage efficiency across these different sections and elevation stages, providing a deeper understanding of the role of drainage in maintaining stability. The proposed methodology can also be successfully applied to other tailings storage facilities, such as tailings dams built downstream or dry stacking piles, contributing to improved monitoring and risk assessment strategies.

## 1. Introduction

The safety of tailings dams has been a growing concern due to the history of accidents with severe environmental, social, and economic consequences [1,2]. In Brazil, the failures of the Fundão Dam (2015) and the Brumadinho Dam (2019) highlighted the vulnerability of these structures and the need to improve monitoring methods and risk management [3]. Statistical studies indicate that failures caused by intense rainfall increased from 25% (before 2000) to 40% (after 2000), while failures associated with poor management rose from 10% to 30%, reinforcing the importance of strict control over dam stability [4]. Additionally, the increase in failure rates is notable across dams of different heights, with a significant rise in collapse rates from 21% to 60% in structures between 15 and 30 m, often linked to rapid construction and inadequate monitoring [4].

The stability analysis of tailings dams requires an effective geotechnical monitoring system before, during, and after their operation. Traditionally, piezometers, settlement plates, inclinometers, water level gauges, and weather stations are used. However, technological advances have introduced new tools, such as radar remote sensors, drones, and robotic total stations, allowing real-time data acquisition [5]. Among these technologies, radar-based remote sensing systems stand out as the most widely used for monitoring sinkholes, displacements, erosion, and subsidence [6,7]. Despite advancements in monitoring technologies, challenges persist in data interpretation, as time series obtained from instrumentation often contain missing measurements, delayed records, and variability in acquisition frequency, making it difficult to establish correlations among instruments. Additionally, data acquired through highly sophisticated and precise monitoring techniques must be validated or compared with datasets of different regularity and accuracy to ensure comprehensive analysis and reliability.

The stability of a dam is directly related to the efficiency of its drainage system, which regulates pore pressure and minimizes the risk of liquefaction. While several studies have analyzed tailings dam failures and structural subsidence worldwide using classical geotechnical techniques and remote sensing, few have focused on cross-correlation analysis of time series to assess the impact of drainage on these failures. Previous works, such as that of Franks et al. (2021), compiled databases of tailings dam failures but did not include detailed statistical modeling of the influence of drainage [8]. Geng et al. (2024) [9], in turn, addressed the stability of a tailings dam considering the effects of infiltration and drainage, using numerical models to simulate the impact of soil compaction and permeability coefficients under different conditions (constant load, precipitation, and drainage). Meanwhile, Li et al. (2023) [10] analyzed the effect of low-permeability layers (mud intercalations) on the stability of infiltration flow in tailings dams by constructing a two-dimensional finite element model, demonstrating that the position and thickness of these layers influence the saturation line and the dam’s safety factor. Other studies present relevant applications of modern tools, such as SAR (Synthetic Aperture Radar), for the monitoring of landslides in Cyprus [11], evaluating subsidence on a highway in China [12], and tracking vertical settlements in the Genzano di Lucania earth dam in Italy [13]. However, these studies do not compare SAR results with other types of classical geotechnical instrumentation, nor do they mention the regularization of time series with missing or faulty data. Additionally, they do not provide information on possible interruptions or failures in data acquisition and storage, or how these issues are handled when they occur. In Turkey (2021), in the Karapınar region, located in the Konya Closed Basin, Orhan et al. [14] analyzed land subsidence and sinkhole activity related to groundwater extraction. These data were correlated through cross-correlations with settlements and horizontal displacements obtained from classical geotechnical instrumentation, including aquifer water level measurements. However, the study does not specify how cross-correlations were obtained or how the different acquisition intervals of the various instrumentation types were considered in the analysis.

In this context, this study proposes an approach based on the interpretation of cross-correlations between geotechnical instrumentation time series to assess the effect of drainage on the stability of tailings dams. The proposed methodology allows for the integration of instrumentation data from different sources and acquisition periods, enabling a more robust analysis of the influence of drainage on the structural behavior of the dam. The novelty of this work lies in the application of advanced statistical models, combining stochastic and inferential techniques for data processing and interpretation.

## 2. Materials and Methods

**Figure 1:**
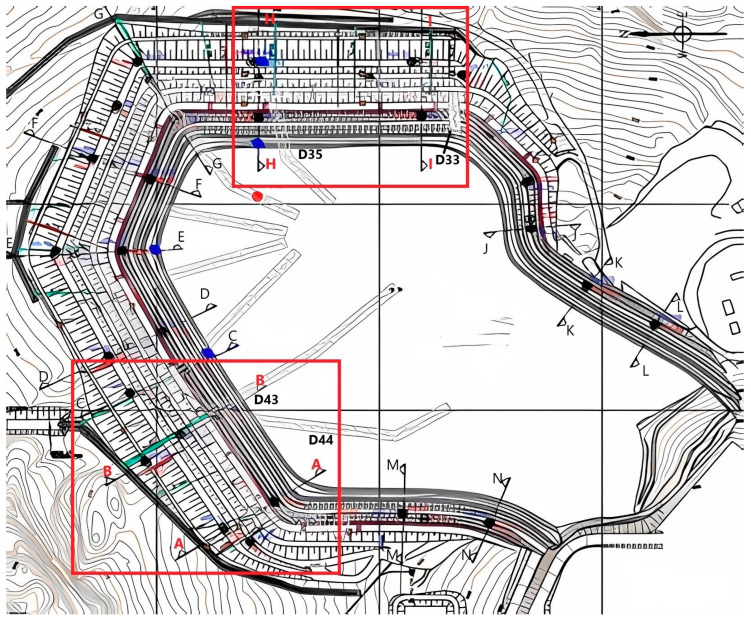
The tailings dam with 14 instrumented sections, with the studied sections (H, I, A, and B) highlighted in red, showing installed instruments (in blue and black) and drains.

To achieve the objectives, the proposed treatment methodology was applied to the monitoring data from four cross-sections (H, I, A, and B) of a tailings dam in Brazil (Figure 1). The dam has been monitored since 2002 through 14 instrumented sections (A to N) and several isolated instruments (Figure 1).

Autocorrelation functions for each instrument and cross-correlations between two instruments were modeled to define the dependence between the variables and their interpretation through drainage and their dependent variables and vice versa.

Due to the importance of drainage in the construction and operation of the dam, the dependence on rainwater, reservoir water, and instruments installed in the solid part of the dam as a function of drainage was modeled and interpreted. Drains were installed at the 2nd elevation stage for sections H and I, and at the 7th elevation stage for sections A and B.

The analysis of a time series requires a comprehensive examination of data recorded over time. This process typically consists of two stages: (i) understanding the structure that generated the series and the dependence between pairs of series (descriptive analysis), and (ii) predicting future values and relationships between series (forecasting models) [15]. Descriptive analysis, which involves modeling the autocorrelation function, is essential for understanding the series’ structure. In contrast, inferential analysis relies on the transfer function to develop mathematical forecasting models. Consequently, time series analysis is traditionally conducted using stochastic models and transfer function models [16,17]. The primary analytical tools for identifying these models are the autocorrelation function for stochastic processes and the cross-correlation between input and output for transfer function models [18].

### 2.1. Dispersion, Asymmetry, Frequency and the Distribution of the Variables Involved

The time series were initially examined using box plot graphs to assess dispersion, asymmetry, and outliers, along with histograms to observe the frequency distribution of the variables.

### 2.2. The Regularization of an Atypical Time Series

A time series consists of numerical data collected at regular intervals over time [19]. However, geotechnical instrumentation monitoring often results in atypical time series, characterized by missing observations, irregular sampling intervals, and lagged data [15]. To enable a comprehensive analysis, it is necessary to estimate the missing values. For the regularization of such atypical time series, Shumway and Stoffer [20] proposed a model using the expectation maximization (EM) algorithm combined with conventional Kalman estimators to estimate parameters based on maximum likelihood [21,22]. Toloi et al. [23] and Hamze-Guilart [15], validated by Alencar et al. [24], addressed this issue by generating daily observations through linear interpolation and subsequently computing weekly averages concentrated on Saturdays, the methodology adopted in this study.

### 2.3. Transformation to a Stationary Time Series

After regularization, the time series must be transformed into a stationary form. A series is considered stationary when its values fluctuate randomly over time around a constant average. The most common approach for achieving stationarity is differentiation, which involves subtracting the previous observation from the current one to create a new series representing the differences between consecutive values (lag-1 difference). This process is repeated iteratively until all temporal dependence is eliminated, i.e., successive differencing is applied to the original series until a stable, stationary dataset is obtained.

### 2.4. Autocorrelation Function

For a regularized stationary series, the autocorrelation function (fac) is used to derive the partial autocorrelation function (facp) of fitting residuals through various operations. The fitting process was performed using an ARIMA (Autoregressive Integrated Moving Average) model. When all lag values fall within the confidence interval, they are considered white noise, indicating the absence of spurious relationships affecting the series. A 5% significance level was adopted for the confidence interval. The autocorrelation function is developed through the following steps: autocorrelation and partial autocorrelation; the autocorrelation and partial autocorrelation of differenced series; and the autocorrelation and partial autocorrelation of residuals from model fitting. The autocorrelation function, applied to a stationary series through different operations, enables the estimation of the partial autocorrelation of residuals, facilitating cross-correlation analysis between two series.

### 2.5. Cross-Correlation Between Two Series

**Figure 2:**
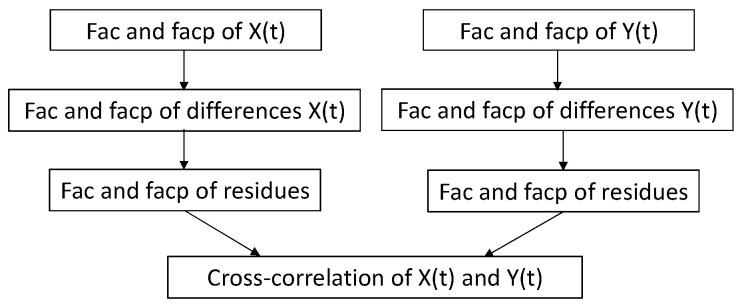
The steps to obtain the cross-correlation between two series [20].

To establish a correlation between two time series, the number of observations and their corresponding dates must be aligned. This requirement is addressed during the regularization process of both series. Figure 2 illustrates the procedure for modeling two time series using autocorrelation functions and cross-correlation analysis between the series.

In this study, the Polihamze statistical programs were used for the regularization of the gross series, while the Gretl software version 1.5.1 was employed for the remaining analyses [15,19].

## 3. Case Study

The tailings dam analyzed in this study is 25 m high and was constructed using the upstream elevation method. It is located in the southeastern region of Brazil, but its identification is omitted due to a confidentiality agreement with the mining company.

This dam was selected for analysis due to the specific stability challenges associated with the upstream elevation method, making it a relevant case for investigations on tailings dam safety. Additionally, it has been continuously monitored since 2002 through an extensive geotechnical instrumentation network, with a variety of instruments distributed across different sections (as illustrated in Figure 1). This long-term monitoring enables a detailed assessment of the spatial and temporal variation in the effects of drainage using time series analysis and cross-correlations.

**Figure 3:**
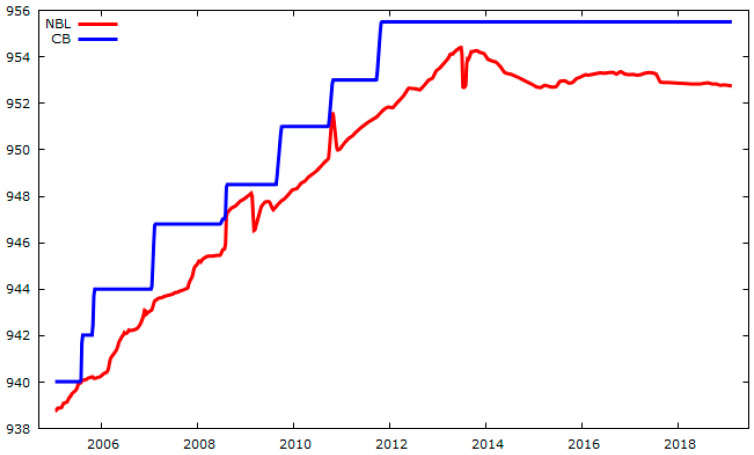
Elevation of dam crest (CB) in m, and reservoir water level (NBL) in m.

The monitoring data were categorized into three phases: (i) during the construction of the starter dam, from the 930 m level to the 940 m level (2002–2005); (ii) upstream elevation from 940 m to 955 m (2005–2011) in seven stages; and (iii) post-deactivation at 955 m in 2011. Table 1 and Figure 3 present the crest elevation and the seven upstream elevations, which were individually analyzed and modeled.

The initial data were provided in Excel worksheets, organized by the installed instrument (variable). Based on the proximity criterion, the data were structured into 14 sections, as shown in Figure 1: 9 instrumented sections (A to I) and 5 intermediate sections (J to N) with individually installed instruments. Additionally, the dataset includes measurements from a rainfall station and reservoir water levels recorded over time.

A total of 26 superficial settlement plates (SMs), dam crest elevation monitoring points (CBs), 44 water level meters (INAs), 41 piezometers (PZs), 10 flow meters in the drains (Ds), reservoir water level gauges (NBLs), and a rain gauge (PLUV) were installed. The settlement plates recorded vertical displacements (MSRs) as well as horizontal movements in the east (MSEs) and north (MSNs) directions.

In this study, the proposed methodology was applied using data from instruments installed in sections H, I, A, and B. These include piezometers (PZs), water level gauges (INAs), and drains (Ds) located near each section, corresponding to instruments numbered 35, 33, 44, and 43, respectively (Figure 1). The period during the dam lift stages was modeled.

Due to the importance of drainage in the construction and operation of the dam, the influence of rainwater and the reservoir water level was analyzed based on data collected from the corresponding instruments—rain gauges (PLUVs) and reservoir water level gauges (NBLs). These data were then modeled and interpreted to assess their relationship with drainage.

Considering its significant impact on the stability of tailings dams, only variables directly associated with drainage were used, either as dependent variables or as influencing factors.

Expression 1 shows the dependent variables and the independent variables.


Y_t_ = B_0_X_t_ + B_1_X_t−1_ + B_2_X_t−2_ + B_n_X_t−n_, (1)

Y—dependent variables;
X—independent variables;
T—time;
B_0_X_t_—a term related to the instantaneous correlation of variables;
B_n_X_t-n_—terms related to significant cross-correlation lags;
n—the lag of cross-correlations.

Table 2 presents the drainage systems of the tailings dam, with the drainages analyzed in this study highlighted in red, indicating their respective sections along with their start and end dates.

Table 3 shows the average rainwater and drainage by dam elevation stage.

The four sections were evaluated by determining cross-correlations between drains 35, 33, 44, and 43 in sections H, I, A, and B, respectively, and the instruments that measure variables related to drainage.

The analysis and discussion of these different sections aim to provide a more comprehensive understanding of the impact of drainage on dam stability. This is achieved through the interpretation of cross-correlations derived from the time series approach, based on historical geotechnical instrumentation data.

## 4. Results and Discussion

All steps for processing the time series, as outlined in item 2, were applied to the selected instruments of the studied sections, including the autocorrelation function and the cross-correlation between input and output [18]. However, to maintain conciseness in the text, only a few illustrative examples of these steps will be presented.

### 4.1. Dispersion, Asymmetry, Frequency, and the Distribution of the Variables Involved

**Figure 4:**
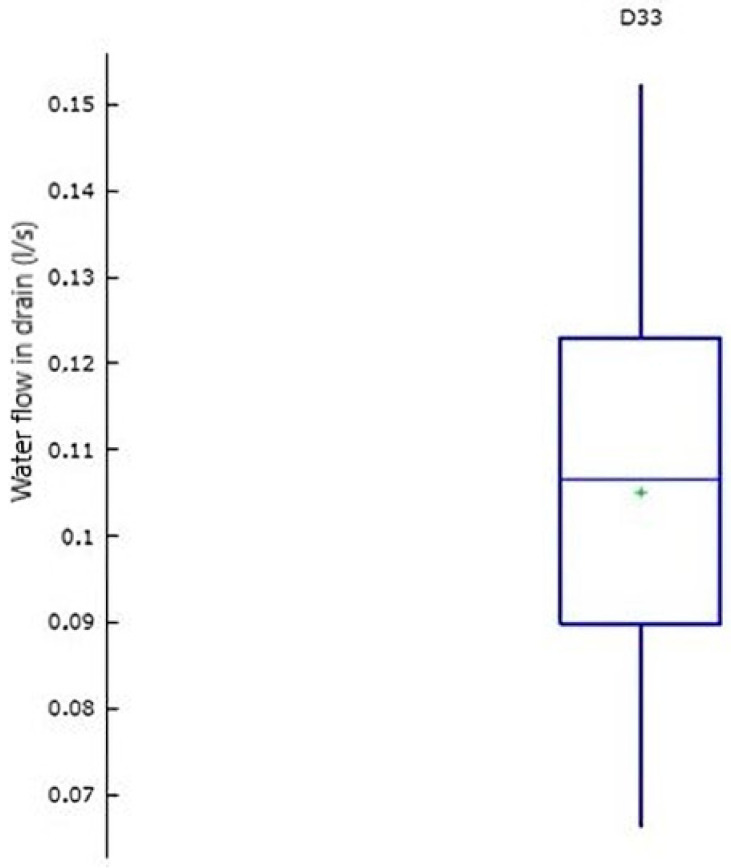
Box plot graphs of drain D33 do not show any discrepancies.

The box plot, as a schematic representation, provides an overview of the position, dispersion, asymmetry, and potential outliers in the data series. Figure 4 presents the box plot for water flows of drain D33 in section I, where no significant discrepancies (outliers) are observed, as there are no values beyond the whiskers indicating their presence. Similarly, the box plots analyzed for other variables also did not reveal any outliers.

**Figure 5:**
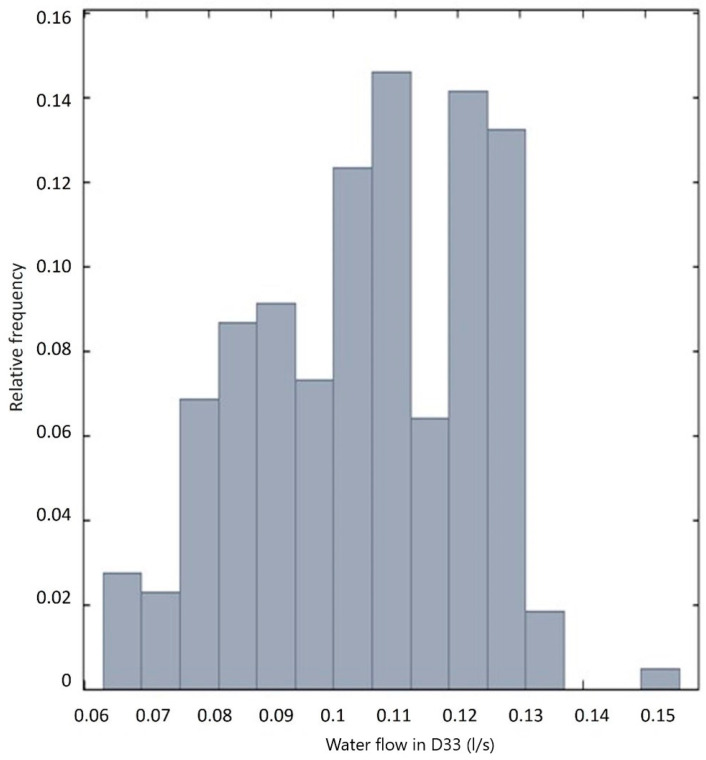
Frequency graphs of drain 33.

Figure 5 shows the histogram of drain D33 in the integrated section I, approaching the Gaussian curve in general. In the histograms of the analyzed variables, some are close to the Gaussian curve; others do not have symmetry, and do not approach any known distribution.

### 4.2. The Regularization of the Time Series

**Figure 6:**
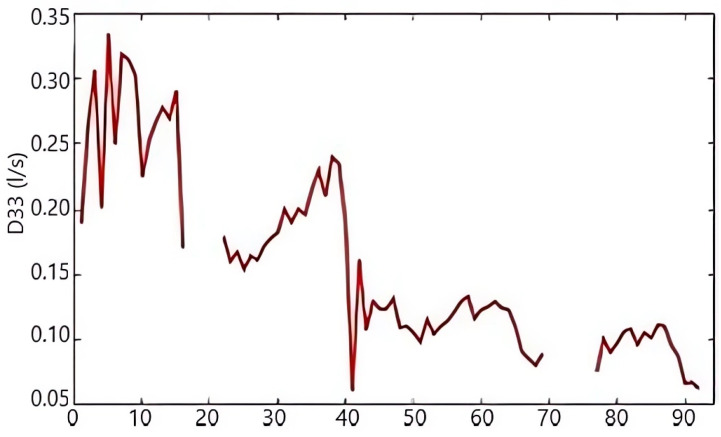
The gross time series of drain D33 during each stage of the dam’s elevation.

**Figure 7:**
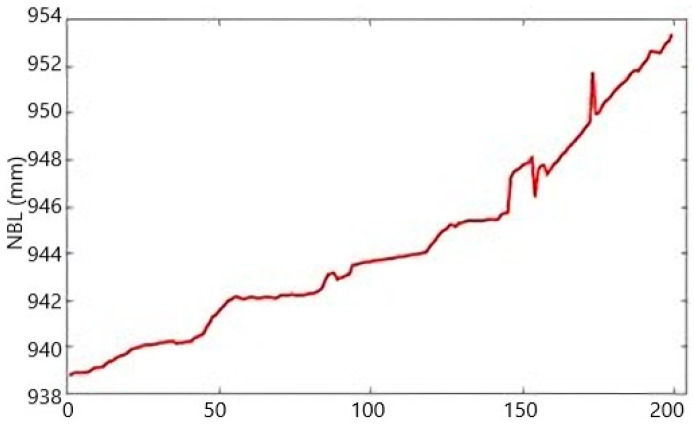
The gross time series of reservoir water level (NBL) during each stage of the dam’s elevation.

**Figure 8:**
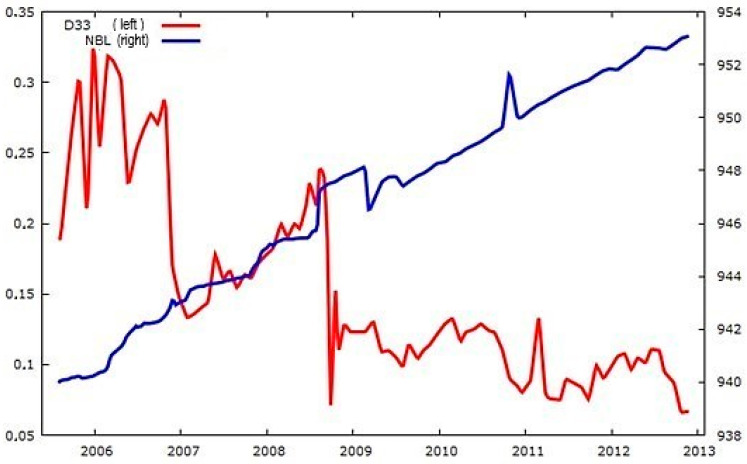
Regularized series D33 and NBL during the dam’s elevation stages; D33 is expressed in L/s and NBL in m.

Figure 6 displays the atypical time series for drain D33 in section I during the dam elevation stage. Between 2005 and 2012, 92 measurements were recorded, with intermittent gaps where no data were collected. During the same period, the water level of the reservoir (NBL) was monitored continuously, with 199 measurements taken without interruptions (Figure 7). However, certain irregularities were identified and corrected through the regularization process, as illustrated in Figure 8. The regularized series, obtained through linear interpolation, is presented in Figure 8.

### 4.3. Transformation to Stationary Time Series

**Figure 9:**
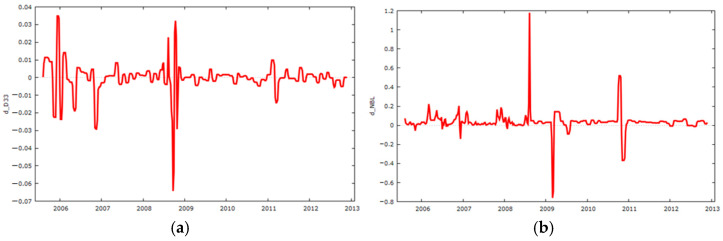
Time series during the elevation stages of the dam transformed into stationary (**a**) D33 and (**b**) NBL.

Figure 9 illustrates the process of transforming the time series of drain D33 and reservoir water level (NBL) into a stationary form during the dam’s elevation stages.

### 4.4. Autocorrelation and Partial Autocorrelation

**Figure 10:**
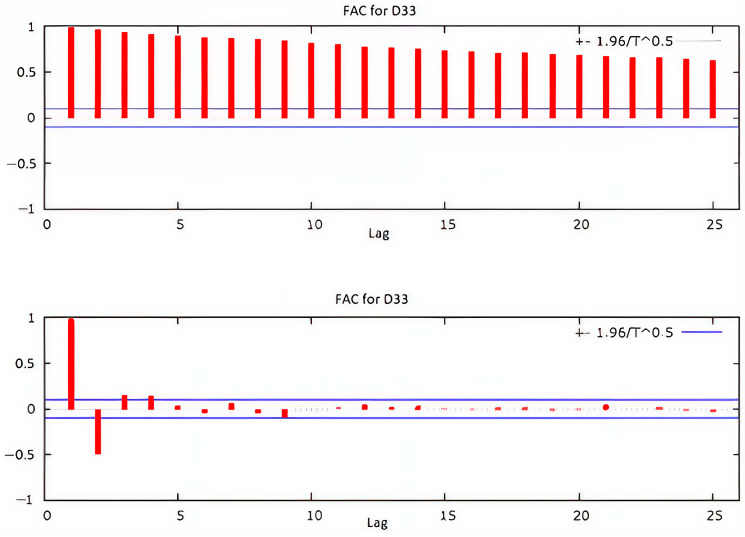
Autocorrelation and partial autocorrelation of drain D33’s time series.

**Figure 11:**
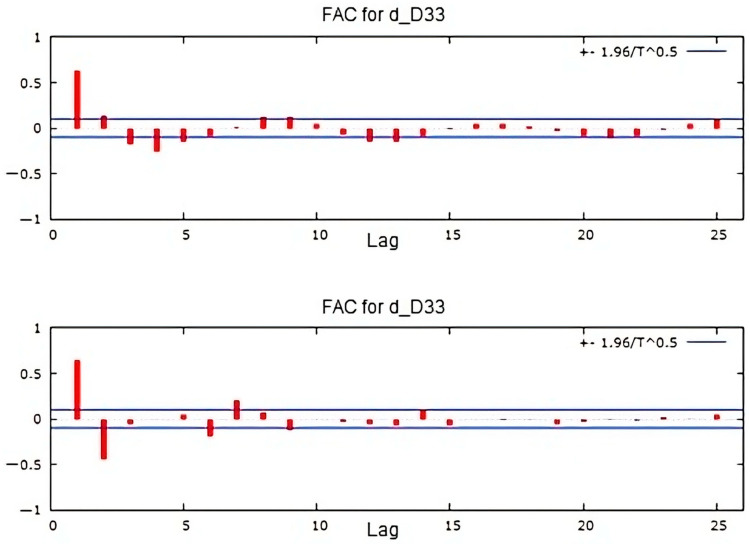
Autocorrelation and partial autocorrelation of the differences in drain D33’s time series.

**Figure 12:**
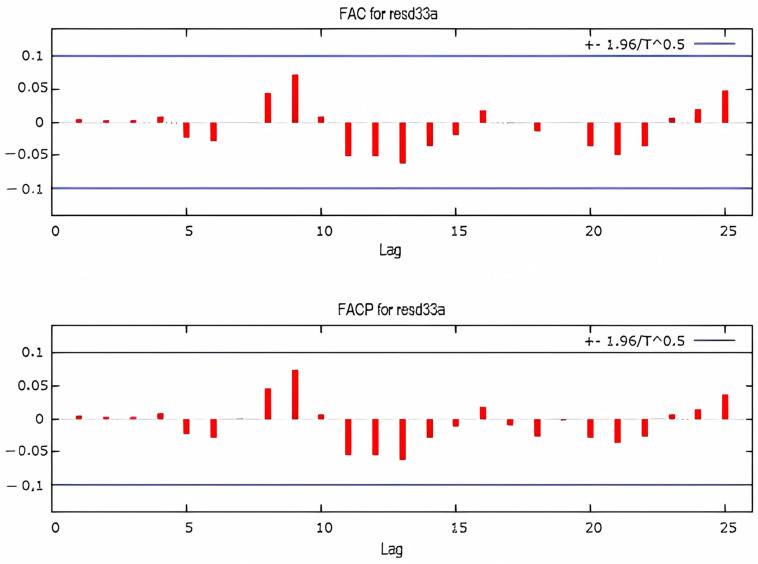
Autocorrelation and partial autocorrelation of fitting residues of drain D33’s time series.

Figure 10 presents autocorrelations (FAC) and partial autocorrelations (FACP) of the time series for drain D33 during the elevation stages of the dam. The horizontal lines define the confidence interval. The series exhibits a decreasing trend, with the first four lags exceeding the confidence interval, while subsequent lags gradually decline toward zero. The partial autocorrelation shows a significant exceedance of the confidence interval for lags 1 and 2. Figure 11 and Figure 12 illustrate the autocorrelation and partial correlation of the differences and the residues, respectively, obtained by fitting with the ARIMA model. In Figure 11, five lags remain outside the confidence interval, whereas in Figure 12, all lags fall within the confidence interval, indicating white noise. As a result, the series can be considered suitable for correlation analysis.

Figure 8 and Figure 9 are primary results and have no direct relationship with cross-correlations. The cross-correlations are modeled from the autocorrelation of the adjusted residuals of two variables, while their relationship is visually represented through scatter plots. Before modeling cross-correlations, scatter plots were analyzed to identify trends and assess potential significant correlations. The positive and negative signs of the cross-correlations indicate the distribution of correlations within the scatter plots.

### 4.5. Cross-Correlations

Table 4 illustrates the cross-correlation of time series for D33 (corresponding to the discharge in drain 33, located near section I, as shown in Figure 1) and NBL (corresponding to the reservoir water level) during the dam’s elevation stages. The positive lags correspond to the cross-correlations of the variables of D33 versus NBL, the negative lags correspond to the cross-correlations of the variables of NBL versus D33. The zero lag is the instant correlation.

Considering that significant correlations are greater than 10%, Table 4 presents five significant correlations (for lags 0, 1, 6, and 7), highlighted in red.

The tailings dam underwent seven upstream elevation stages (Table 1). Thus, cross-correlations between monitoring instruments in these sections, starting from the second elevation phase of the dam, were modeled.

In sections H and I of the dam, drainage was carried out from the second elevation stage to the seventh elevation stage; in sections A and B, it was only carried out in the seventh elevation stage.

Table 5 shows the cross-correlations during the second elevation stage in section H utilizing drain D35 and the variables PLUV (rain gauge), NBL (reservoir water level), INA8H (water level meter 8H), and INA14H (water level meter 14H) to assess the drainage effect.

Table 6 displays the cross-correlation during the second elevation stage in section I using drain D33 and the variables PLUV (rain gauge), NBL (reservoir water level), INA15I (water level meter 15I), PZ6I (piezometer 6I), and PZ11I (piezometer 11I) for the drainage effect evaluation.

In both section H (Table 5) and section I (Table 6), drainage begins in the second elevation stage. In both cases, significant instantaneous cross-correlations predominantly occur, indicating the rapid response of the instruments at the onset of drainage.

Table 5 highlights relevant instantaneous cross-correlations between drain D35 and the reservoir water level (NBL), between D35 and the water level meter 8H (INA8H), and between D35 and the water level meter 14H (INA14H). In section H, apparently, the drain, piezometers, and water level meter inside the dam were responding to the water accumulated in the reservoir rather than to precipitation. This behavior is likely due to water accumulation during the first elevation stage, when the drainage system had not yet been implemented (Table 3). Table 6, on the other hand, shows significant instantaneous cross-correlations between drain D33 and the rain gauge (PLUV), in addition to those between D33 and the water level meter 15I (INA15I), as well as between D33 and the piezometers PZ6I and PZ11I. Drain D33 was probably releasing water from rainfall as well as from the reservoir. Additionally, in both sections, the lagged cross-correlations maintain a consistent pattern in terms of intensity and frequency, suggesting balanced drainage in both sections despite differences in the variables considered.

Table 7 and Table 8 present the cross-correlations among the monitoring instruments in sections H and I, respectively, during the third elevation stage.

In section I, during the third elevation stage (Table 8), instantaneous correlations predominate, except for the PLUV vs. D33 correlations and vice versa. The significant lagged cross-correlations occur at minimal frequencies and exhibit medium to low intensities, suggesting stability in this part of the dam.

In section H (Table 7), both instantaneous and lagged cross-correlations are minimal in both intensity and frequency, which may indicate that drainage has been more effective in this section, contributing to greater overall stability compared to section I.

As shown in Table 3, drained flux at this elevation stage was 0.36 L/s in section H and 0.17 L/s in section I, further supporting this interpretation.

Table 9 illustrates the cross-correlation during the fourth elevation stage in section H, utilizing, in addition to the instruments already mentioned in previous elevation stages—namely, drain D35, PLUV, NBL, INA8H, and INA14H (Table 5 and Table 7)—the INA26H (water level meter 26H), PZ5H (piezometer 5H), and PZ26H (piezometer 26H) to assess the drainage effect.

Table 10 presents the cross-correlations between the monitoring instruments in section I during the fourth elevation stage.

In the fourth elevation stage, in section H (Table 9), instantaneous correlations do not predominate, indicating that drainage in this stage was very effective. In contrast, in section I (Table 10), instantaneous correlations are present but with low values, suggesting that drainage was also effective in this stage. Notably, the lagged cross-correlations exhibit lower intensity and frequency compared to the second elevation stage in both sections, which may indicate that the dam became dehydrated due to drainage. This same trend is generally observed in the third elevation stage for both sections H and I. As shown in Table 3, from the second elevation stage onward, drainage gradually decreases until the fourth elevation stage in both sections, further supporting this interpretation.

Table 11 presents the cross-correlations between the monitoring instruments in section H during the fifth elevation stage. In addition to the instruments already mentioned in previous elevation stages, namely, drain D35, PLUV, NBL, INA8H, INA14H, INA26H, PZ5H, and PZ26H (Table 9), the water level meter 9H (INA9H) was considered to assess the drainage effect.

Table 12 presents the cross-correlations between the monitoring instruments in section I during the fifth elevation stage. In addition to the instruments already mentioned in previous elevation stages, namely, drain D33, PLUV, NBL, INA15I, PZ26I (Table 6, Table 8 and Table 10), the water level meter 27I (INA27I) was considered to assess the drainage effect.

In the fifth elevation stage, section I (Table 12) exhibits a different behavior to section H, as there are no significant instantaneous correlations except between D35 and PLUV and vice versa. This may be associated with the higher average precipitation recorded during this stage (126.6 mm) compared to 80.1 mm in the fourth elevation stage, as shown in Table 3. The difference observed between section H (Table 11) and section I (Table 12) suggests that section H was more saturated with water, leading to more intense drainage.

Table 13 and Table 14 present the cross-correlations among the monitoring instruments in sections H and I, respectively, during the sixth elevation stage.

In the sixth elevation stage, section H (Table 13), there are no significant instantaneous correlations between drain D35 and the NBL and PLUV variables. However, significant correlations are observed between D35 and the water level meters (INA8H, INA9H, and INA14H), as well as between D35 and the piezometer PZ5H. Additionally, the presence of significant lagged correlations suggests that drainage is more associated with accumulated water in the dam rather than with rainfall-induced inflows, due to the lack of precipitation during this stage. As shown in Table 3, rainfall was lower in the sixth elevation stage compared to the fifth and seventh elevation stages.

In the sixth elevation stage, section I (Table 14), in general, maintains the same behavior as section H, which may also be the result of smaller precipitation of water in this stage than in the previous one.

Table 15 and Table 16 illustrate the cross-correlations among the monitoring instruments in sections H and I, respectively, during the seventh elevation stage.

In sections H and I of the seventh elevation stage (Table 15 and Table 16) there are practically no instantaneous correlations, and the significant lagged correlations are of low frequencies and intensities, which shows that the dam is very stable, despite this being the rainiest period, according to Table 3, with 135.5 mm on average.

As explained in item 2, during the seventh elevation stage, in addition to sections H and I, drainage was also carried out in sections A and B (Figure 3). Apparently, the drainage in these two sections starts in these stages as these two sections are more distant from the recycling water dam than sections H and I, where drainage started in the second elevation stage (Figure 3).

Table 17 illustrates the cross-correlation during the seventh elevation stage in section A utilizing drain D44 and the variables PLUV (rain gauge), NBL (reservoir water level), INA10A (water level meter 10A), and INA19A (water level meter 19A) to assess the drainage effect.

Table 18 presents the cross-correlation during the seventh elevation stage phase in section B utilizing drain D43 and the variables PLUV (rain gauge), NBL (reservoir water level), INA3B (water level meter 3B), INA20B (water level meter 20B), PZ29B (piezometer 29B), and PZ20B (piezometer 20B) to evaluate the drainage effect.

In section A (Table 17) at the seventh elevation stage, there are significant instantaneous correlations between D44 and NBL, and between D44 and INA19 A, and the significant lagged correlations are well balanced, suggesting that the drainage is related to water inside the reservoir and inside the dam.

In section B (Table 18), significant instantaneous correlations prevail between drain D43 and the water level meters (INA3B and INA20B), as well as between D43 and the piezometers (PZ20B and PZ29B). However, there are no significant instantaneous correlations between drainage and the NBL or PLUV variables, which may indicate that, in this section, drainage is not influenced by rainwater or the reservoir but rather by water confined within the dam. It is observed that the lagged cross-correlations are of the same intensities as the instantaneous drainage correlations and the INA and PZ variables, also the frequencies of the lagged cross-correlations are relatively stable. In the case of drainage and the NBL and PLUV variables, the significant lagged cross-correlations are of low frequency and intensity, which confirms that water release is fundamentally due to the interstitial water of the dam.

As shown by the analysis of the results presented in Table 15 and Table 16, which examine the cross-correlations in sections H and I, respectively, the tailings dam in its seventh elevation stage can be considered highly stable, even during the rainiest period, with an average precipitation of 135.5 mm (Table 3).

This stability is largely attributed to the intense drainage carried out during this pre-decommissioning stage, not only in sections A and B but also in other areas not covered in this study (Table 2). Notably, drainage rates in sections A and B reached 0.79 L/s and 0.86 L/s, respectively (Table 3), the highest recorded values.

## 5. Conclusions

This study demonstrates the relevance of the approach based on the interpretation of cross-correlations between geotechnical and hydrological variables obtained by instrumentation along the elevation stages of a tailings dam to assess the evolution and efficiency of the drainage system and understand its influence on the stability of the dam.

The results indicate that drainage efficiency varies both over time and between different sections of the dam, as reflected in the response of monitoring instruments. It was also possible to track the contribution of rainfall, accumulated water in the reservoir, and interstitial water in the dam to the response of piezometers, the water level, and the drains.

During the seventh elevation stage, which recorded the highest average precipitation (135.5 mm), the dam remained stable, with few significant instantaneous correlations and lagged correlations of low frequency and intensity. This result suggests that the drainage system was effective in controlling water infiltration and dam saturation, minimizing the increase in pore water pressure, thereby reducing the risks of instability before pre-decommissioning. The recorded drainage flow rates in sections A and B during the seventh elevation stage (0.79 L/s and 0.86 L/s, respectively) were the highest in the historical series.

The processing, analysis, and modeling of historical geotechnical instrumentation data through time series, combined with the geotechnical interpretation of cross-correlations, proved to be an effective tool for assessing the impact of drainage on dam stability. Cross-correlations allow inferences regarding the direction, intensity, and frequency of rainwater and water contained in the tailings, as well as the hydration and dehydration processes of the dam over time due to drainage.

The results obtained can, therefore, contribute to improving geotechnical monitoring strategies and assisting in decision-making regarding drainage management in tailings dams. Furthermore, the approach used can be adapted for application in other tailings storage systems, such as dry stacking piles, enabling a more comprehensive assessment of the safety of these structures.

The treatment of atypical instrumentation series and the subsequent interpretation of cross-correlations can be implemented in AI and machine learning processes in order to speed up safety assessment and decision-making in tailings dams or piles construction.

## Figures and Tables

**Table 1:** Height and time of the seven elevations.

Elevation Stage	Initial Level (m)	Final Level (m)	Initial Date	Final Date
1	940	942	15 January 2005	13 August 2005
2	942	944	13 August 2005	12 November 2005
3	944	947	12 November 2005	12 July 2008
4	947	949	12 July 2008	5 September 2009
5	949	951	5 September 2009	18 September 2010
6	951	953	18 September 2010	10 September 2011
7	953	955	10 September 2011	9 December 2012

**Table 2:** Discharge in drain.

Discharge in Drain	Section	Initial Date	Final Date
D06	C	20 September 2005	22 November 2007
D33	I	5 August 2005	20 February 2015
D34	F/G	5 August 2005	17 June 2018
D35	H	20 September 2005	17 June 2018
D36	G/H	23 March 2011	17 June 2016
D37	G	20 September 2005	14 June 2018
D39	E/F	29 June 2009	17 June 2016
D41	D/E	23 February 2011	18 August 2017
D43	B	6 August 2005	14 August 2019
D44	A	5 August 2005	14 August 2019

**Table 3:** Average rain gauge measurements and drainage flows at each elevation stage.

Elevation Stage	Rain Gauge (mm)	Water Flow in Drain (L/s)			
		D35 Section H	D33 Section I	D44 Section A	D43 Section B
1	90.2	0	0	0	0
2	106.0	0.48	0.27	0	0
3	119.1	0.36	0.17	0	0
4	80.1	0.17	0.12	0	0
5	126.6	0.14	0.13	0	0
6	105.4	0.37	0.09	0	0
7	135.5	0.47	0.09	0.79	0.86

**Table 4:** Cross-correlations between D33 and NBL throughout the entire dam elevation process.

Lag	Cross-Correlation D33 vs. NBL	Lag	Cross-Correlation NBL vs. D33
0	0.163 ^1^	0	0.163
1	−0.113	−1	−0.096
2	−0.024	−2	0.021
3	−0.049	−3	0.012
4	−0.063	−4	0.031
5	0.029	−5	−0.009
6	−0.179	−6	0.048
7	−0.247	−7	−0.004

^1^ Values in red represent significant correlations (>10%). Positive and negative signs represent the position of the cross-correlation in the scatter plot.

**Table 5:** Cross-correlations for section H—2nd elevation stage. Weekly: full range—13 August 2005 to 12 November 2005.

Series	Lag: Cross-Correlation							
	0	1	2	3	4	5	6	7
PLUV vs. D35 ^1^	0.013	0.058	−0.075	0.040	−0.040	0.038	−0.114 ^2^	0.084
D35 vs. PLUV	0.013	0.056	−0.031	−0.041	0.032	−0.017	0.058	0.102
NBL vs. D35	−0.118	0.081	−0.040	0.082	−0.023	0.175	−0.086	−0.012
D35 vs. NBL	−0.118	0.004	−0.039	−0.043	0.022	−0.046	−0.046	0.019
INA8H vs. D35	0.223	0.002	0.054	0.131	0.033	−0.284	−0.017	−0.168
D35 vs. INA8H	0.223	0.004	0.016	−0.024	−0.022	0.243	−0.249	0.102
INA14H vs. D35	0.104	0.188	−0.046	0.036	−0.061	−0.051	0.015	−0.094
D35 vs. INA14H	0.104	−0.201	0.059	0.068	0.044	−0.430	0.450	−0.135

^1^ D35—drain 35; PLUV—rain gauge; NBL—reservoir water level; INA8H—water level meter 8H, INA14H—water level meter 14H. ^2^ Values in red represent significant correlations (>10%). Positive and negative signs represent the position of the cross-correlation in the scatter plot.

**Table 6:** Cross-correlations for section I—2nd elevation stage. Weekly: full range—13 August 2005 to 12 November 2005.

Series	Lag: Cross-Correlation							
	0	1	2	3	4	5	6	7
PLUV vs. D33 ^1^	−0.113 ^2^	−0.340	−0.156	0.158	−0.030	0.032	−0.017	0.000
D33 vs. PLUV	−0.113	0.077	0.001	0.053	0.161	−0.110	0.073	0.051
NBL vs. D33	−0.096	−0.045	−0.098	−0.154	0.097	0.241	−0.035	−0.055
D33 vs. NBL	−0.096	−0.036	−0.005	0.004	−0.031	−0.080	−0.004	−0.027
INA15I vs. D33	−0.245	−0.061	−0.054	0.072	0.221	−0.072	−0.024	−0.183
D33 vs. INA15I	−0.245	0.184	0.026	−0.105	0.097	0.323	−0.114	−0.007
PZ6I vs. D33	−0.488	−0.083	0.012	0.057	0.042	0.089	0.025	0.093
D33 vs. PZ6I	−0.488	0.033	0.041	−0.086	0.037	0.128	−0.034	−0.007
PZ11I vs. D33	0.176	0.101	−0.175	0.051	−0.004	−0.136	−0.003	0.170
D33 vs. PZ11I	0.176	−0.027	−0.131	−0.082	−0.061	0.006	−0.156	0.149

^1^ D33—drain 33; PLUV—rain gauge; NBL—reservoir water level; INA15I—water level meter 15I; PZ6I—piezometer 6I; PZ11I—piezometer 11I. ^2^ Values in red represent significant correlations (>10%). Positive and negative signs represent the position of the cross-correlation in the scatter plot.

**Table 7:** Cross-correlations for section H—3rd elevation stage. Weekly: full range—12 November 2005 to 12 July 2008.

Series	Lag: Cross-Correlation							
	0	1	2	3	4	5	6	7
NBL vs. D35 ^1^	0.068	−0.014	−0.012	0.063	0.078	−0.065	−0.082	0.066
D35 vs. NBL	0.068	0.193 ^2^	0.010	−0.089	0.001	−0.058	0.011	−0.082
PLUV vs. D35	−0.020	−0.160	0.114	−0.009	0.014	−0.002	0.258	−0.058
D35 vs. PLUV	−0.020	−0.027	0.091	−0.099	−0.026	−0.002	0.078	−0.238
INA8H vs. D35	0.133	0.050	0.054	−0.002	−0.072	0.030	−0.19	−0.030
D35 vs. INA8H	0.133	−0.015	−0.020	0.047	−0.033	−0.006	−0.020	0.004
INA14H vs. D35	−0.082	−0.050	0.028	0.032	0.084	0.056	−0.009	0.054
D35 vs. INA14H	0.000	0.016	0.024	−0.001	−0.068	0.020	0.017	0.037

^1^ D35—drain 35; PLUV—rain gauge; NBL—reservoir water level; INA8H—water level meter 8H, INA14H—water level meter 14H. ^2^ Values in red represent significant correlations (>10%). Positive and negative signs represent the position of the cross-correlation in the scatter plot.

**Table 8:** Cross-correlations for section I—3rd elevation stage. Weekly: full range—12 November 2005 to 12 July 2008.

Series	Lag: Cross-Correlation							
	0	1	2	3	4	5	6	7
PLUV vs. D33 ^1^	0.006	−0.183 ^2^	−0.012	0.039	0.062	0.012	−0.057	0.153
D33 vs. PLUV	0.006	−0.005	−0.041	−0.102	0.145	−0.020	0.011	−0.037
NBL vs. D33	−0.113	0.078	−0.050	−0.081	0.075	−0.078	0.024	0.021
D33 vs. NBL	0.113	0.050	−0.006	−0.008	−0.008	−0.007	−0.061	0.005
INA15I vs. D33	−0.173	0.013	−0.030	0.061	0.069	0.116	0.030	0.047
D33 vs. INA15I	−0.173	0.013	−0.030	0.061	0.069	0.116	0.030	0.047
PZ6I vs. D33	−0.133	−0.023	0.032	0.029	−0.035	−0.045	0.124	0.034
D33 vs. PZ6I	−0.133	−0.018	−0.030	−0.024	−0.017	0.053	−0.033	0.097
PZ11I vs. D33	−0.414	−0.060	0.078	0.026	0.070	0.068	0.010	0.045
D33 vs. PZ11I	−0.414	0.069	0.029	0.069	−0.065	0.256	0.000	0.143

^1^ D33—drain 33; PLUV—rain gauge; NBL—reservoir water level; INA15I—water level meter 15I; PZ6I—piezometer 6I; PZ11I—piezometer 11I. ^2^ Values in red represent significant correlations (>10%). Positive and negative signs represent the position of the cross-correlation in the scatter plot.

**Table 9:** Cross-correlations for section H—4th elevation stage. Weekly: full range—12 July 2008 to 5 September 2009.

Series	Lag: Cross-Correlation							
	0	1	2	3	4	5	6	7
PLUV vs. D35 ^1^	−0.010	0.012	−0.265 ^2^	0.109	0.043	0.016	−0.059	−0.049
D35 vs. PLUV	−0.010	0.022	0.016	0.080	−0.033	−0.064	−0.024	0.043
NBL vs. D35	0.138	−0.030	−0.025	0.009	−0.071	−0.013	−0.006	0.007
D35 vs. NBL	0.138	−0.060	0.058	−0.045	0.081	−0.023	0.049	−0.050
INA8H vs. D35	−0.012	0.043	−0.249	−0.069	−0.066	−0.074	0.066	0.048
D35 vs. INA8H	−0.012	−0.005	−0.008	−0.117	−0.012	−0.048	0.028	0.030
INA14 vs. D35	−0.015	−0.274	−0.084	−0.027	0.053	−0.062	−0.047	−0.127
D35 vs. INA14	−0.015	0.096	−0.012	−0.359	0.041	0.057	0.224	0.330
IN26H vs. D35	0.068	0.080	0.103	0.008	0.176	−0.484	−0.091	−0.040
D35 vs. INA26H	0.068	−0.011	−0.044	−0.056	−0.010	−0.018	−0.012	0.009
PZ5H vs. D35	0.014	−0.058	−0.093	−0.081	0.049	−0.110	0.047	−0.038
D35 vs. PZ5H	0.014	0.058	0.052	0.062	0.065	0.019	0.068	0.041
PZ26H vs. D35	0.191	−0.123	−0.173	−0.070	−0.172	0.476	0.210	0.061
D35 vs. PZ26H	0.191	0.071	0.183	0.103	0.037	0.002	0.014	0.024

^1^ D35—drain 35; PLUV—rain gauge; NBL—reservoir water level; INA8H—water level meter 8H, INA14H—water level meter 14H; INA26H—water level meter 26H; PZ5H—piezometer 5H; PZ26H—piezometer 26H. ^2^ Values in red represent significant correlations (>10%). Positive and negative signs represent the position of the cross-correlation in the scatter plot.

**Table 10:** Cross-correlations for section I—4th elevation stage. Weekly: full range—12 July 2008 to 5 September 2009.

Series	Lag: Cross-Correlation							
	0	1	2	3	4	5	6	7
PLUV vs. D33 ^1^	0.040	0.048	−0.042	0.020	−0.013	0.071	−0.089	−0.108 ^2^
D33 vs. PLUV	0.040	0.022	0.016	0.080	−0.033	−0.064	−0.024	0.043
NBL vs. D33	0.217	0.080	−0.003	−0.021	−0.008	−0.011	−0.001	−0.003
D33 vs. NBL	0.217	−0.123	−0.020	0.039	−0.160	−0.010	−0.315	−0.471
INA15I vs. D33	−0.149	0.069	0.084	−0.114	0.152	−0.128	0.027	0.021
D33 vs. INA15I	0.149	−0.147	−0.167	0.094	0.469	0.123	0.475	0.171
PZ6I vs. D33	0.103	−0.054	−0.029	−0.026	−0.029	0.001	0.060	0.021
D33 vs. PZ6I	0.103	0.027	0.125	0.013	−0.014	0.048	0.077	0.082
PZ11I vs. D33	−0.206	−0.026	0.029	−0.026	−0.018	0.001	−0.010	0.008
D33 vs. PZ11I	−0.206	0.135	−0.026	0.183	0.215	0.220	0.629	0.263

^1^ D33—drain 33; PLUV—rain gauge; NBL—reservoir water level; INA15I—water level meter 15I; PZ6I—piezometer 6I; PZ11I—piezometer 11I. ^2^ Values in red represent significant correlations (>10%). Positive and negative signs represent the position of the cross-correlation in the scatter plot.

**Table 11:** Cross-correlations for section H—5th elevation stage. Weekly: full range—5 September 2009 to 18 September 2010.

Series	Lag: Cross-Correlation							
	0	1	2	3	4	5	6	7
PLUV vs. D35 ^1^	−0.195 ^2^	0.083	0.048	0.072	−0.044	−0.176	0.128	0.087
D35 vs. PLUV	−0.195	0.038	0.057	−0.170	−0.038	0.019	0.079	0.149
NBL vs. D35	−0.520	−0.054	0.032	0.165	0.173	0.158	0.021	−0.006
D35 vs. NBL	−0.520	−0.024	0.106	−0.047	−0.011	0.137	0.062	−0.002
INA8H vs. D35	0.188	−0.005	0.023	−0.098	−0.351	0.029	0.026	−0.113
D35 vs. INA8H	0.188	−0.028	0.034	0.048	−0.032	−0.126	0.032	−0.119
INA9H vs. D35	0.258	−0.039	−0.071	0.011	0.284	−0.005	−0.059	0.160
D35 vs. INA9H	0.000	−0.011	−0.071	−0.198	−0.116	0.114	0.004	−0.191
INA14H vs. D35	0.466	0.009	0.047	−0.112	−0.152	−0.066	−0.032	0.000
D35 vs. INA14H	0.466	0.065	−0.026	−0.102	0.213	−0.009	−0.076	−0.127
INA26H vs. D35	0.494	−0.003	0.022	−0.140	0.034	0.145	−0.066	0.270
D35 vs. PZ26H	0.494	−0.154	0.062	0.013	−0.385	0.062	0.001	0.116
PZ5H vs. D35	−0.227	−0.037	0.036	0.294	0.146	0.004	−0.004	0.285
D35 vs. PZ5H	−0.227	0.156	−0.026	−0.116	0.028	0.089	−0.064	−0.008
PZ26H vs. D35	−0.548	0.078	−0.013	0.270	0.308	−0.130	0.034	0.086
D35 vs. PZ26H	−0.548	−0.154	0.062	0.013	−0.385	0.062	0.001	0.116

^1^ D35—drain 35; PLUV—rain gauge; NBL—reservoir water level; INA8H—water level meter 8H, INA14H—water level meter 14H; INA26H—water level meter 26H; INA9H—water level meter 9H; PZ5H—piezometer 5H; PZ26H—piezometer 26H. ^2^ Values in red represent significant correlations (>10%). Positive and negative signs represent the position of the cross-correlation in the scatter plot.

**Table 12:** Cross-correlations for section I—5th elevation stage. Weekly: full range—5 September 2009 to 18 September 2010.

Series	Lag: Cross-Correlation							
	0	1	2	3	4	5	6	7
PLUV vs. D33 ^1^	−0.267 ^2^	−0.246	0.083	0.068	−0.043	0.113	−0.051	−0.020
D33 vs. PLUV	−0.267	0.056	−0.127	0.225	0.455	−0.088	0.118	0.037
NBL vs. D33	0.065	−0.067	0.061	0.048	−0.096	−0.071	−0.088	−0.004
D33 vs. NBL	0.065	0.064	−0.062	0.010	−0.299	0.033	0.215	−0.088
INA15I vs. D33	0.022	0.023	−0.015	0.106	0.051	0.118	0.007	−0.078
D33 vs. INA15I	0.022	0.047	0.022	−0.059	0.018	0.240	0.030	0.091
PZ6I vs. D33	0.031	0.132	0.064	0.142	−0.045	0.087	0.153	−0.139
D33 vs. PZ6I	0.031	−0.019	0.138	−0.059	0.038	−0.067	0.031	−0.038
INA27I vs. D33	0.022	0.023	−0.015	0.105	−0.051	0.118	0.007	−0.078
D33 vs. INA27I	0.022	0.047	0.022	0.059	0.018	0.240	0.030	0.091

^1^ D33—drain 33; PLUV—rain gauge; NBL—reservoir water level; INA15I—water level meter 15I; INA27I—water level meter 27I; PZ6I—piezometer 6I. ^2^ Values in red represent significant correlations (>10%). Positive and negative signs represent the position of the cross-correlation in the scatter plot.

**Table 13:** Cross-correlations for section H—6th elevation stage. Weekly: full range—18 September 2010 to 10 September 2011.

Series	Lag: Cross-Correlation							
	0	1	2	3	4	5	6	7
PLUV vs. D35 ^1^	−0.039	−0.238 ^2^	−0.066	0.078	0.001	0.299	0.101	−0.165
D35 vs. PLUV	−0.039	0.030	−0.128	0.089	0.050	0.049	0.034	−0.248
NBL vs. D35	0.013	0.934	0.014	−0.002	0.006	0.034	0.024	0.026
D35 vs. NBL	0.013	0.038	−0.043	−0.051	−0.087	0.040	−0.020	−0.161
INA8H vs. D35	−0.307	0.029	−0.056	−0.080	0.337	0.017	−0.051	−0.001
D35 vs. INA8H	−0.307	0.058	0.116	0.007	0.103	0.463	0.001	0.013
INA9H vs. D35	−0.100	0.018	−0.134	−0.069	−0.075	−0.087	−0.014	0.082
D35 vs. INA9H	−0.100	−0.065	0.136	0.006	0.012	−0.275	−0.160	−0.143
INA14H vs. D35	0.150	0.052	−0.041	−0.052	−0.135	−0.059	−0.108	−0.133
D35 vs. INA14H	0.150	0.047	0.094	−0.033	0.070	0.652	−0.022	−0.136
INA26H vs. D35	−0.066	0.018	−0.050	−0.092	−0.109	−0.049	−0.092	0.039
D35 vs. INA26H	−0.066	−0.025	0.091	−0.104	−0.012	0.498	−0.116	−0.129
PZ5H vs. D35	−0.372	−0.035	−0.010	−0.023	0.165	−0.093	−0.007	0.094
D35 vs. PZ5H	−0.372	−0.162	0.016	0.069	0.356	0.736	−0.040	−0.047
PZ26H vs. D35	−0.082	0.025	0.006	−0.043	0.036	−0.043	−0.060	−0.011
D35 vs. PZ26H	−0.082	0.040	0.031	−0.069	0.082	−0.188	−0.217	0.368

^1^ D35—drain 35; PLUV—rain gauge; NBL—reservoir water level; INA8H—water level meter 8H, INA14H—water level meter 14H; INA26H—water level meter 26H; PZ5H—piezometer 5H; PZ26H—piezometer 26H. ^2^ Values in red represent significant correlations (>10%). Positive and negative signs represent the position of the cross-correlation in the scatter plot.

**Table 14:** Cross-correlations for section I—6th elevation stage. Weekly: full range—18 September 2010 to 10 September 2011.

Series	Lag: Cross-Correlation							
	0	1	2	3	4	5	6	7
PLUV vs. D33 ^1^	−0.091	−0.234 ^2^	−0.054	0.042	0.014	0.283	−0.005	−0.078
D33 vs. PLUV	−0.091	−0.045	−0.179	0.119	0.020	0.023	0.029	−0.144
NBL vs. D33	−0.163	0.026	0.022	−0.027	0.004	0.057	−0.014	−0.004
D33 vs. NBL	−0.163	−0.043	0.025	0.014	−0.031	−0.108	−0.103	−0.214
INA15I vs. D33	−0.246	0.137	0.078	0.010	0.127	−0.302	0.024	0.079
D33 vs. INA15	−0.246	−0.108	0.094	0.042	0.073	0.728	−0.062	−0.074
PZ6I vs. D33	−0.080	0.124	−0.026	−0.043	−0.030	−0.124	0.106	0.142
D33 vs. PZ06	−0.080	−0.110	0.064	0.023	0.003	0.743	−0.001	−0.114
INA27I vs. D33	0.037	−0.015	0.009	0.043	−0.165	0.055	−0.026	−0.089
D33 vs. INA27I	0.037	0.038	−0.025	0.167	0.097	−0.230	0.078	0.206

^1^ D33—drain 33; PLUV—rain gauge; NBL—reservoir water level; INA15I—water level meter 15I; INA27I—water level meter 27I; PZ6I—piezometer 6I. ^2^ Values in red represent significant correlations (>10%). Positive and negative signs represent the position of the cross-correlation in the scatter plot.

**Table 15:** Cross-correlations for section H—7th elevation stage. Weekly: full range—10 September 2011 to 9 December 2012.

Series	Lag: Cross-Correlation							
	0	1	2	3	4	5	6	7
PLUV vs. D35 ^1^	0.081	−0.240 ^2^	−0.016	−0.003	0.089	−0.013	0.234	−0.116
D35 vs. PLUV	0.081	0.030	−0.128	0.089	0.050	0.049	0.034	−0.248
NBL vs. D35	−0.403	0.105	0.044	0.032	0.106	0.212	−0.004	0.155
D35 vs. NBL	−0.403	0.050	0.072	−0.027	0.060	0.016	0.076	0.000
INA8H vs. D35	−0.013	−0.079	−0.034	−0.274	−0.122	−0.178	−0.025	−0.202
D35 vs. INA8H	−0.013	−0.104	−0.083	−0.061	0.050	0.170	−0.005	−0.029
INA26H vs. D35	−0.012	0.032	0.049	0.063	0.069	−0.052	0.026	−0.051
D35 vs. INA26H	−0.012	−0.100	0.042	−0.021	0.052	−0.057	0.007	−0.016
PZ5H vs. D35	−0.018	−0.025	−0.021	−0.026	−0.053	0.001	−0.016	0.014
D35 vs. PZ5H	−0.018	−0.036	−0.035	−0.033	−0.023	−0.001	−0.013	−0.041
PZ26H vs. D35	0.076	0.007	−0.051	0.053	−0.093	0.018	−0.134	−0.572
D35 vs. PZ26H	0.076	0.030	0.045	0.006	−0.004	0.021	−0.011	−0.032

^1^ D35—drain 35; PLUV—rain gauge; NBL—reservoir water level; INA8H—water level meter 8H, INA26H—water level meter 26H; PZ5H—piezometer 5H; PZ26H—piezometer 26H. ^2^ Values in red represent significant correlations (>10%). Positive and negative signs represent the position of the cross-correlation in the scatter plot.

**Table 16:** Cross-correlations for section I—7th elevation stage. Weekly: full range—10 September 2011 to 9 December 2012.

Series	Lag: Cross-Correlation							
	0	1	2	3	4	5	6	7
PLUV vs. D33 ^1^	0.016	0.405 ^2^	−0.185	0.063	−0.079	−0.099	−0.228	0.000
D33 vs. PLUV	0.016	0.096	0.135	0.215	−0.146	−0.089	−0.111	0.037
NBL vs. D33	−0.166	0.062	−0.007	−0.092	0.099	0.005	0.015	0.022
D33 vs. NBL	−0.166	−0.047	−0.026	0.140	−0.090	−0.130	0.069	0.107
INA15I vs. D33	0.102	0.031	0.080	0.023	−0.021	0.091	−0.053	−0.109
D33 vs. INA15I	0.102	0.121	−0.033	0.004	0.010	0.145	0.026	0.055
PZ6I vs. D33I	−0.024	0.070	0.008	0.046	−0.026	0.251	−0.001	−0.022
D33 vs. PZ06	−0.024	0.142	−0.010	0.064	0.047	0.016	0.018	0.006

^1^ D33—drain 33; PLUV—rain gauge; NBL—reservoir water level; INA15I—water level meter 15I; PZ6I—piezometer 6I. ^2^ Values in red represent significant correlations (>10%). Positive and negative signs represent the position of the cross-correlation in the scatter plot.

**Table 17:** Cross-correlations for section A—7th elevation stage. Weekly: full range—10 September 2011 to 9 December 2012.

Series	Lag: Cross-Correlation							
	0	1	2	3	4	5	6	7
PLUV vs. D44 ^1^	0.057	−0.074	−0.010	−0.028	0.048	−0.166 ^2^	0.081	0.023
D44 vs. PLUV	0.057	0.004	0.146	−0.418	−0.166	−0.029	0.099	0.302
NBL vs. D44	0.161	−0.011	−0.049	−0.030	−0.024	0.138	0.025	0.070
D44 vs. NBL	0.161	−0.050	−0.037	−0.148	−0.251	−0.309	−0.012	−0.148
INA10A vs. D44	0.002	0.006	0.020	0.126	−0.041	−0.041	0.057	0.131
D44 vs. INA10A	0.002	0.249	0.021	0.045	0.056	−0.018	0.018	0.047
INA19A vs. D44	−0.196	0.084	0.121	−0.005	−0.059	0.063	−0.064	−0.018
D44 vs. INA19A	−0.196	0.129	0.236	−0.072	0.200	0.462	−0.044	−0.025

^1^ D44—drain 44; PLUV—rain gauge; NBL—reservoir water level; INA10A—water level meter 10A; INA19A—water level meter 19A. ^2^ Values in red represent significant correlations (>10%). Positive and negative signs represent the position of the cross-correlation in the scatter plot.

**Table 18:** Cross-correlations for section B—7th elevation stage. Weekly: full range—10 September 2011 to 9 December 2012.

Series	Lag: Cross-Correlation							
	0	1	2	3	4	5	6	7
NBL vs. D43 ^1^	0.048	−0.158 ^2^	0.079	0.048	0.207	−0.074	0.036	−0.018
D43 vs. NBL	0.000	0.027	0.041	0.092	0.150	−0.004	−0.100	−0.338
PLUV vs. D43	−0.090	−0.260	0.035	0.049	0.030	0.134	0.254	−0.090
D43 vs. PLUV	0.000	−0.042	−0.204	0.201	−0.035	0.084	−0.033	0.272
INA3B vs. D43	−0.296	0.049	−0.064	0.269	−0.110	0.048	0.024	0.012
D43 vs. INA3B	−0.296	0.049	−0.064	0.269	−0.110	0.048	0.024	0.000
INA20B vs. D43	−0.344	−0.216	−0.074	0.167	−0.039	0.190	0.053	0.005
D43 vs. INA20B	−0.344	−0.216	−0.074	0.167	−0.039	0.190	0.053	0.005
PZ20B vs. D43	0.148	−0.018	−0.059	0.015	−0.201	−0.031	0.005	0.028
D43 vs. PZ20B	0.148	−0.018	−0.059	0.015	−0.201	−0.031	0.005	0.028
PZ29B vs. D43	0.146	−0.019	−0.005	−0.037	−0.244	0.037	0.017	0.025
D43 vs. PZ29B	0.146	0.027	0.010	−0.086	0.202	0.098	0.133	0.001

^1^ D43—drain 43; PLUV—rain gauge; NBL—reservoir water level; INA3B—water level meter 3B; INA20B—water level meter 20B; PZ29B—piezometer 29B; PZ20B—piezometer 20B. ^2^ Values in red represent significant correlations (>10%). Positive and negative signs represent the position of the cross-correlation in the scatter plot.

## Data Availability

Data were obtained from the mining company and are available from the authors only with the permission of the mining company.
